# Supplementary material for: Urine Untargeted Metabolomic Profiling Is Associated with the Dietary Pattern of Successful Aging among Malaysian Elderly
Source: Nutrients. 2020 Sep 23;12(10):2900. doi: 10.3390/nu12102900 (PMC7597952; doi:10.3390/nu12102900)
Supplement: Supplementary file 1 [file nutrients-12-02900-s001.pdf]

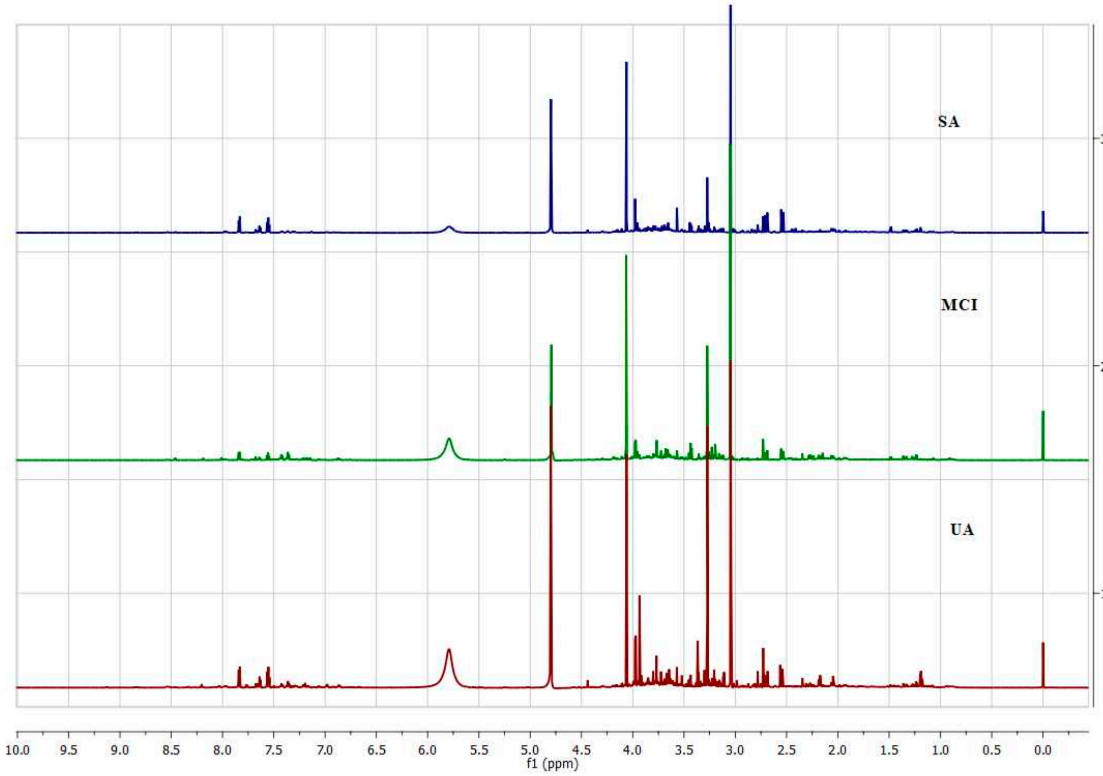

Figure S1 ^{1}H-NMR spectra (600 MHz) of urine for SA, MCI and UA representatives

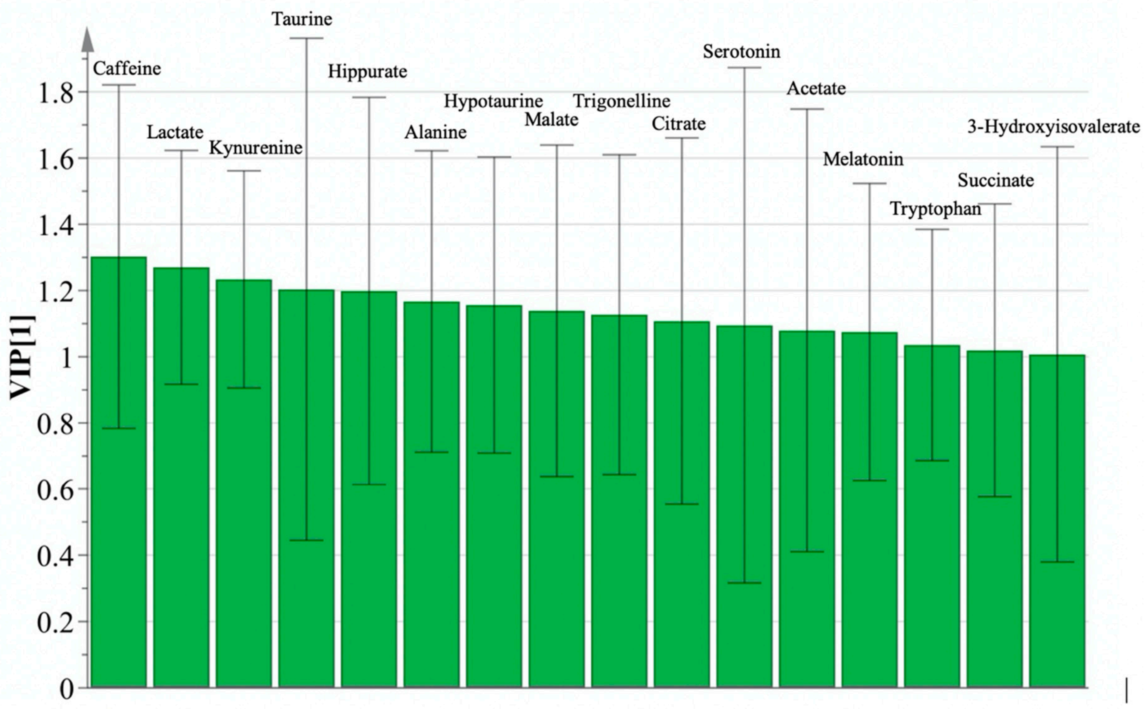

Figure S2 Variable importance of projection for PLS-DA model

Component	R2X	R2X(cum)	Eigenvalue	R2Y	R2Y(cum)	Q2	Limit	Q2(cum)	Significance	Iterations
0	Cent.									
1	0.503	0.503	13.6	0.384	0.384	0.352	0.05	0.352	R1	7
2	0.111	0.613	2.99	0.253	0.637	0.227	0.05	0.499	R1	6

Table S1 Summary of fit for PCA model
